# Structural aspects of the inactive X chromosome

**DOI:** 10.1098/rstb.2016.0357

**Published:** 2017-09-25

**Authors:** Giancarlo Bonora, Christine M. Disteche

**Affiliations:** 1 Department of Genome Sciences, University of Washington, Seattle, WA 98195, USA; 2 Department of Pathology, University of Washington, Seattle, WA 98195, USA; 3 Department of Medicine, University of Washington, Seattle, WA 98195, USA

**Keywords:** X chromosome, X inactivation, chromatin, nucleus

## Abstract

A striking difference between male and female nuclei was recognized early on by the presence of a condensed chromatin body only in female cells. Mary Lyon proposed that X inactivation or silencing of one X chromosome at random in females caused this structural difference. Subsequent studies have shown that the inactive X chromosome (Xi) does indeed have a very distinctive structure compared to its active counterpart and all autosomes in female mammals. In this review, we will recap the discovery of this fascinating biological phenomenon and seminal studies in the field. We will summarize imaging studies using traditional microscopy and super-resolution technology, which revealed uneven compaction of the Xi. We will then discuss recent findings based on high-throughput sequencing techniques, which uncovered the distinct three-dimensional bipartite configuration of the Xi and the role of specific long non-coding RNAs in eliciting and maintaining this structure. The relative position of specific genomic elements, including genes that escape X inactivation, repeat elements and chromatin features, will be reviewed. Finally, we will discuss the position of the Xi, either near the nuclear periphery or the nucleolus, and the elements implicated in this positioning.

This article is part of the themed issue ‘X-chromosome inactivation: a tribute to Mary Lyon’.

## 1. Introduction

Mammalian X-chromosome inactivation (XCI) results in the random silencing of one of the two X chromosomes in females early in development in order to ensure a balance in the dosage of X-linked gene expression between the sexes [1]. The process of XCI is initiated by the upregulation of an X-linked long non-coding RNA (lncRNA), *XIST* (in the human context, or *Xist* in mouse), triggered, at least in part, by the downregulation of pluripotency factors during embryogenesis and by specific activator elements [2,3]. While significant differences exist between mammalian species in terms of the timing and events associated with the onset of XCI, one unifying theme is that *XIST* RNA molecules ultimately coat the inactive X chromosome (Xi) *in* *cis* and recruit chromatin modifiers that lead to the silencing of most of the genes along its length [4–6]. A lot of research has been focused on gaining a better understanding of the mechanisms behind XCI and the structural nature of the Xi, not only because it is inherently interesting, but it also serves as a useful model for understanding gene regulation in general, and has implications for various disease states. The process of XCI and the central role of the lncRNA *XIST* have been previously reviewed and are further addressed in other articles in this issue [6–9]. Here, we will specifically review efforts to address the Xi's exceptional architecture within the nucleus. First, we will briefly summarize key seminal findings and ongoing research based on imaging approaches. Second, we will present findings regarding the three-dimensional (3D) structure of the Xi made using orthogonal biochemical methods that leverage the power of high-throughput DNA sequencing. Third, we will consider the position of the Xi within the nucleus.

## 2. Historical perspective

Mammalian X-chromosome structure has long been the subject of fascination, speculation and vigorous study. In 1949, Barr and Bertram [10] first reported a body in the nucleus of feline nerve cells that was susceptible to Nissl staining, which they designated a ‘nucleolar satellite’. What was particularly intriguing about this nuclear body was that they only saw it within the neurons of female cats, but not within those of male cats. This led them to conjecture that it may well be ‘derived from the heterochromatin of the sex chromosomes’—specifically excess chromosomal material that might arise from the X chromosome being duplicated in females [10]. This ‘Barr body’ was subsequently found to exist in a number of different mammalian cell types, but always exclusively within female cells [11,12]. A decade passed before Ohno & Hauschka [13] deduced that only one of the two X chromosomes exhibited the particular structure. This led Mary Lyon to hypothesize shortly thereafter that the distinctive structure of the Barr body was related to its function, or rather lack thereof, in that it was a manifestation of an inactivated X chromosome with respect to expression, with its homologue being in a transcriptionally active state (Xa) [1,13].

Ohno & Hauschka [13] speculated that the Barr body's singular structure possibly reflected the fact that it was more compact than its homologue. However, it was unclear whether this perceived compactness of the Xi relative to the Xa, and to all autosomes, was due to the former having a reduced volume relative to the latter, or whether this impression was simply due to differences in their relative shapes, surface areas and chromatin character [9]. A series of studies emanating from the laboratory of the Cremer brothers led efforts to clarify this question. Three-dimensional confocal fluorescence microscopy performed on female cells sourced from amniotic fluid showed that, in terms of volume, the Xa was not substantially larger than the Xi (approx. 1.2×) [14]. The authors noted that this ratio fell well within the mean difference seen between the volumes of larger and smaller chromosome 1 homologues in males (approx. 1.5×). In contrast, the surface area of the Xa was appreciably greater than that of the Xi (approx. 1.9×), indicative of a difference in their respective shapes [14]. In follow-up studies on the same cell type, the group generated 3D reconstructions of the X chromosomes based on serial sections obtained with a confocal laser scanning microscope, which confirmed that the X homologues shared relatively similar volumes, but that the Xa did indeed exhibit a larger, more irregular, surface area in contrast with the Xi's apparently smoother surface and rounder shape [15,16]. Quantitative 3D multicolour fluorescence *in situ* hybridization (FISH) in human diploid fibroblasts provided additional evidence for a smooth spherical Xi, in contrast to a more irregular ellipsoidal Xa with a large surface area [17].

What is unequivocal is that the silencing of genes along the Xi initiated by the spreading of *XIST* RNA is accompanied by a depletion of RNA polymerase II and of active chromatin marks such as histone acetylation and histone H3 methylation at lysine 4 (H3K4me2), and by an enrichment of repressive chromatin features such as histone H3 methylation at lysine 27 (H3K27me3) and histone H2A ubiquitination at lysine 119 (H2AK119u). These initial epigenetic changes are followed by accumulation of the histone variant macroH2A, DNA methylation at CpG islands of X-linked genes and later deposition of histone H3 methylation at lysine 9 (H3K9me3) and histone H4 methylation at lysine 20 (H4K20me3) [6,18–23]. The accumulation of these repressive epigenetic attributes results in the Xi's heterochromatic character and contributes to its distinctive appearance in images that rely on fluorescent DNA probes for FISH and protein antibodies for immunostaining. Early imaging studies revealed that facultative (H3K27me3, H2AK119u and macroH2A) and constitutive (H3K9me3) heterochromatin marks appear mutually exclusive on the human Xi, resulting in a succession of bands along the chromosome [22,24]; such a non-overlapping pattern was also observed in mouse based on recent ChIP-seq profiles [25]. However, profiling of histone modifications in human indicates that the two repressive histone marks are not completely exclusive, but do overlap in some regions, depending on the cell type [26,27]. The uneven distribution of heterochromatic marks along the Xi suggests that its 3D compaction may also be uneven. Rego *et al.* [28] used both light and electron microscopy on mouse and human fibroblasts to argue that the Xi does indeed possess a very particular heterochromatic character that is neither euchromatic nor constitutively heterochromatic in nature, but rather more akin to the tightly packed heterochromatin found in prophase chromatids. Notably, they observed that unlike uniformly dense centromeric heterochromatin, the Xi appears to have space running through dense regions. Uneven compaction of the Xi was also observed by measuring distances between X segments labelled with different colours; in this model, channels of less condensed chromatin surround clusters of condensed silent chromatin [17].

Recent work taking advantage of the latest technological advances in imaging used super-resolution 3D structured illumination microscopy to provide additional evidence for non-uniform Xi chromatin compaction [29]. Although structural features such as chromatin domains of preferential interaction (potentially corresponding to topological associating domains discussed below) and the channels that pervade these domains were observed in both X homologues, previously transcriptionally permissive active regions appeared to have at least partially collapsed following XCI [29,30]. This suggests that there is indeed a certain level of Xi compaction as Ohno & Hauschka [13] initially conjectured.

## 3. Three-dimensional structure of the inactive X chromosome in mammals

Chromosome conformation capture (3C) has provided researchers with a powerful alternative tool to study the 3D conformation of chromosomes in the nucleus [31,32]. With this protocol, formaldehyde cross-linking and proximity ligation is followed by restriction endonuclease digestion to produce a 3C library of protein-mediated DNA–DNA interactions within an ensemble of cells. The presence of hybrid sequences representing interactions can then be assayed using polymerase chain reaction (PCR) with primers for sequences representing loci of putative interaction. The advent of massively parallel or high-throughput DNA sequencing (HTS), in conjunction with the availability of genomic assemblies for many organisms, has revolutionized the ability of researchers to interrogate biological structure and function, allowing for this to be done on a genome-wide scale, with many protocols having been developed to leverage this paradigm-shifting technology [33]. 4C-seq (Circular 3C coupled to HTS) adds a round of digestion and ligation to the 3C protocol, which results in the generation of a library of circular ligation products that theoretically represent DNA–DNA contacts with a particular locus of interest (the bait or viewpoint) within the population of cells [34]. The DNA fragments representing the regions that interact with the viewpoint can be amplified from the hybrid DNA library by means of inverse PCR using primers for the bait locus, which are then subjected to HTS. Using the 4C-seq method in a mouse system where reads could be assigned to either the Xa or the Xi based on single-nucleotide polymorphisms (SNPs), Splinter *et al*. [35] showed that silenced genes on the Xi make far fewer long-range *cis*-contacts than do genes on the Xa, where active genes interact with other active regions. Those genes on the Xi that escape XCI, however, appear to form contacts with other escapee genes.

In addition to 4C-seq, which allows one to generate interaction profiles between one locus of interest and all other loci in the genome, 3C-based approaches leveraging HTS have also been developed that allow one to determine interaction frequencies between any pair of loci in the genome from which heat maps of interaction frequency (contact maps) can be produced. For instance, chromosome conformation capture carbon copy (5C) uses a series of primer pairs arrayed along a stretch of the genome to obtain contact maps representing interactions between all loci within a region of interest [36]. Nora *et al.* [30] employed the 5C method to investigate the 3D structure of the mouse X chromosome. More specifically, they determined the contact frequencies within a 4.5 Mb region around the *Xist* locus in mouse embryonic stem cells (ESCs) and discovered that this region consisted of a series of discrete 200 kb–1 Mb regions that showed preferential interactions, which they termed topologically associating domains (TADs). TADs were observed both before and after ESC differentiation to neuronal progenitor cells (NPCs) and in male primary mouse embryonic fibroblasts (MEFs). Furthermore, in the differentiated cells, TADs were retained by both the Xa and the Xi based on super-resolution 3D DNA-FISH and the deconvolution of female MEF 5C data into contributions from each X chromosome. However, TADs were attenuated along the Xi presumably due to the fact that XCI leads to a reduction in these intra-TAD contact frequencies.

The Hi-C protocol extends the 5C method and theoretically permits one to determine the contact frequencies between all DNA–DNA interactions, at least for mappable regions of the genome, by essentially multiplexing 4C-seq across every locus of interest. However, very deep sequencing is typically required to obtain reasonable levels of coverage and resolution, especially in the case of mammalian genomes [37,38]. Briefly, Hi-C bridges interacting DNA fragments along with a biotin, which can be selected with streptavidin beads to produce a library representing a sampling of all protein-mediated DNA–DNA contacts. Results based on Hi-C data published by Dixon *et al*. [39] concurrently with the aforementioned paper by Nora *et al.* [30] showed that TADs were not specific to the region around *Xist*/*XIST*, but were, in fact, a conserved hallmark of chromatin structure and observed to occur along all chromosomes, both in mouse and human. It should also be noted that the size of TADs is similar to that of interconnected chromosomal regions previously described as chromatin domain clusters (CDCs) based on observations made using microscopy studies, although no formal correspondence has been shown to exist between TADs and CDCs [40,41].

Hi-C libraries were originally prepared using genomic material liberated from nuclei, but more recently the protocol has been applied to intact nuclei (*in situ* Hi-C) with the assumption that this would capture the chromosomal interactions more faithfully. By sequencing *in situ* Hi-C libraries for human B-lymphoblastoid cells (GM12878) to an unprecedentedly deep level, Rao *et al.* [42] were able to produce contact maps specific for both the maternal and paternal chromosomes using SNPs on GM12878. Although intrachromosomal contact maps for autosomal homologues were largely similar, pronounced differences were observed between the maps for the Xa and the Xi (typically the paternal X chromosome in GM12878). While the Xa contact map showed all the hallmarks seen for autosomes (e.g. compartments and TADs) [37,39], the Xi lacked these features and instead appeared to be partitioned into two large ‘superdomains’ with the boundary midway along the long arm of the human X chromosome [42]. This boundary region contains the macrosatellite repeat locus *DXZ4*, which encodes an lncRNA and is conserved across mammals. Interestingly, *DXZ4* had been hypothesized to play a role in X-chromatin organization because the chromatin insulator CTCF, a zinc finger protein essential for chromatin organization, was found to bind at the locus specifically on the Xi [43,44]. Chadwick's group and others also showed that, on the Xi, *DXZ4* has a euchromatic, hypomethylated character, as opposed to the locus on the Xa, which is heterochromatic and methylated [43–45]. Using their high-resolution Hi-C data, Rao *et al*. [42] showed that the human Xi, but not the Xa, features a series of ‘superloops’ of interaction anchored at 24 X-linked loci. Among these loci were the lncRNA loci *XIST*, *DXZ4*, *FIRRE* and *ICCE* (loc550643), the latter three of which consist of tandem repeats that are euchromatic in character and bind CTCF only in the Xi [44]. Indeed, all but one of the 24 superloop anchor points were determined to exist along the Xi-bound CTCF, confirming the factor's critical role in 3D conformation of nuclear DNA.

**Figure 1.**
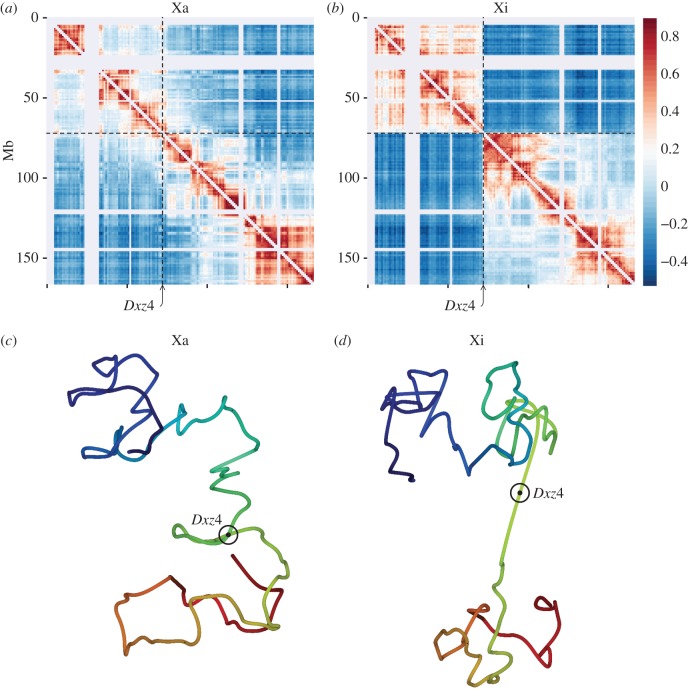
(*a*,*b*) Allelic intrachromosomal contact maps reveal the bipartite structure of the Xi in mouse F_1_ brain tissue (spretus Xi). (*a*) A heat map of the Pearson correlation of contact counts within 1 Mb windows along the Xa obtained from BL6-specific *in situ* DNase Hi-C reads shows compartments and TAD structures typically found in autosomes. (*b*) As in (*a*) but for spretus-specific reads showing the two superdomains of preferential interaction along the Xi. (*c*,*d*) Three-dimensional models inferred from the contact maps in (*a*) and (*b*), respectively, provide a visualization of the two distinct X-chromosome structures. The location of the *Dxz4* locus (within the hinge region) is indicated by the ringed point. The red to blue shading runs from the centromere to telomere. (Adapted from [47].)

*In situ* DNase Hi-C is a variant of the Hi-C protocol that uses DNase to digest DNA rather than relying on restriction endonucleases that depend on specific cut sites, thereby improving coverage and resolution [46]. Using this method on a highly polymorphic F_1_ hybrid mouse system (BL6 × spretus) with skewed XCI both *in vivo* (whole brain with a spretus Xi) and *in vitro* (Patski cells with a BL6 Xi), Deng and co-workers produced allelic contact maps that revealed that the Xi possessed less defined TADs relative to the Xa, as had also been observed in mouse fibroblasts (figure 1*a*,*b*) [47,48]. In their place were found two superdomains, demonstrating a bipartite structure of the mouse Xi (figure 1*b*). Three-dimensional models inferred from the DNase Hi-C data allowed for the definition of a hinge between the superdomains (figure 1*c*,*d*) [47]. Similar to the human Xi, the hinge on the mouse Xi was found to harbour the conserved *Dxz4* macrosatellite repeat locus, but also the minisatellite repeat *Ds-TR* not found in human [49,50]. While the overall bipartite structure of the human and mouse Xis is conserved, the relative size and content of the superdomains differ between species, with the mouse hinge located centrally, whereas the human hinge is located more distally on the long arm [47]. Unlike the situation in human, superloops between *Dxz4*, *Firre* and *Xist* were not detected in two studies done in mouse cells [47,51], but were detected in a third study [52], which could reflect differences in cell types, methodologies and/or low frequency of events. RNA-FISH for *Xist* in multiple mouse cell types (female primary neurons, embryonic fibroblasts and Patski cells) appeared to confirm that the Xi was coated by the lncRNA in two separate regions, separated by a single *Dxz4* DNA-FISH signal, though this was only found to be the case in less than 10% of nuclei, possibly due to limitations of two-dimensional FISH and/or the loss of structural integrity caused by the FISH procedure [47]. Taken together, these results support the observation made by Ohno & Hauschka [13] that ‘the sex chromatin often seems clearly bipartite’ in chromatin spreads of mouse cells.

Additional validation of the Xi bipartite structure has come by way of Wang *et al.* [53], who recently introduced a multiplexed FISH method that enabled 3D tracing of the central positions of 40 of 86 X-chromosome TADs, as defined using combined Hi-C data from both X chromosomes. One thousand different primary oligonucleotide (30 nt) probes were used to target the central 100 kb region of each TAD, with histone variant macroH2A immunostaining being used to distinguish the Xi from the Xa. Averaging over multiple experiments, the mean spatial distance between each pair of TADs was used to construct pairwise distance matrices (akin to Hi-C contact matrices) for each homologue. Those for the Xi and the Xa were found to be strikingly different, with Xi matrix elements being more homogeneous and exhibiting smaller values than for Xa matrix. A power law function fitted to a plot of spatial versus genomic distance for the Xa produced a scaling exponent similar to that seen for autosomes (approx. 1/5), whereas the Xi yielded a far smaller scaling factor (approx. 1/13). This, the authors argue, suggests that the Xi is more compact and possesses a more intermixed chromatin arrangement with homogeneous distances, similar to that observed for Polycomb-repressed domains [54].

To investigate the role of the hinge in the Xi bipartite structure, Giorgetti *et al*. [51] used CRISPR/cas9 editing to remove an approximately 200 kb region incorporating the *Dxz4* locus from the Xi allele of an F1 hybrid female mouse ESC line (129 × Cast), with XCI completely skewed towards the 129 homologue. Allele-specific Hi-C was performed on clones following differentiation to NPCs, which revealed that the Xi bipartite structure was disrupted in the deletion mutant. Despite the large reorganization of Xi structure observed in the mutant line, XCI establishment was seemingly largely unaffected. One rather counterintuitive result was noted: a subset of facultative escape genes showed reduced escape in certain NPC clones, as confirmed by accessibility and expression analyses. However, this reduced potential for escape was variable among different NPC clones, suggesting that it may not be related to the hinge deletion. In a separate experiment, induction of wild-type *Xist* expression (but not of a mutant *Xist* lacking the A-repeat) from an inducible locus in male ESCs was shown to be sufficient to promote the formation of two superdomains in the sole X chromosome [51].

The disruption to Xi structure upon allelic removal of the *Dxz4*-harbouring hinge region observed in the mouse was recapitulated in the human Xi by Darrow *et al*. [52], who deleted a large 300 kb region containing the *DXZ4* locus in a differentiated retinal pigment epithelial cell line (RPE1). This resulted in the loss of the superdomains, as well as loss of the Xi-specific *DXZ4*-*FIRRE* superloop, but not of the *FIRRE-ICCE* superloop, suggesting that the superloops form independently of each other. An equivalent deletion to Xa had no such effect. The loss of Xi bipartite structure in the mutant coincided with the disruption of an interaction compartment (as reflected by principal eigenvectors) in the vicinity of the *DXZ4* locus. This interval also saw a switch in its chromatin state (from H3K27me3 to H3K9me3 enrichment) and replication timing status (from early to late) [52]. Such a large change in the structure of the Xi following deletion of the hinge raised the expectation of possible reactivation of silenced genes; however, similarly to what was reported in mouse ES cells [51], the authors saw little change with respect to phenotype and gene expression in the human cells, which may reflect the multiple layers of regulatory control of silencing of the Xi.

## 4. Location of specific elements

The Xi is not completely silenced and certain X-linked genes escape XCI. As hypothesized by Mary Lyon as early as 1962, some of these genes fall within ‘a short pairing segment, that is not normally inactivated’, which has come to be known as the pseudoautosomal region (PAR) [8,55,56]. PARs show homology with the Y chromosome and are involved in sex chromosome meiotic pairing, as seen for autosomal homologues, with genes within these regions behaving like most autosomal genes in that they show levels of expression that are relatively even [57,58]. The chromatin configuration of the PAR apparently differs from the rest of the condensed Xi in that the distal short arm of the human X often retains a euchromatic uncondensed configuration, as shown in abnormal duplicated X chromosomes [59].

In addition to PAR genes, other genes on the Xi, not necessarily having Y-linked counterparts, are also known to escape inactivation in proportions that differ between species and tissue types [60–63]. Based on a re-analysis of data aggregated from three previously published studies, Balaton *et al*. [63] estimate that 12–20% genes escape XCI in humans. By contrast, Berletch *et al.* [62] reported that a smaller proportion of genes were found to escape XCI in mouse tissues (3–7%). However, in an *in vitro* system using an embryonic kidney-derived Patski F1 hybrid mouse cell line, the same study reported seeing a larger set of escape genes (21%) that included both kidney- and cell line-specific escapees. Although escape genes were found to be distributed all along the mouse Xi, they tended to co-localize with CTCF-binding clusters, suggesting a potential role for CTCF binding in demarcating regions of escape and inactivation and in the compartmentalization of the Xi in general [62]. Interestingly, Patski cells which exhibit a higher proportion of escape than *in vivo* samples, also showed a relatively low density of CTCF peaks. The relative paucity of CTCF may cause them to have a more relaxed structure in the inactivated state, allowing for an expansion of escape domains [62]. This ties in with previous studies, indicating that CTCF plays a role in the segregation of silenced and escape domains [64,65], with 3C-based results showing that chromatin interaction features such as domains and loops are often delineated by CTCF together with cohesin [39,42,66], as well with a recent result showing that regions that exhibit stable tendencies to escape XCI coincide with TADs observed in ESCs [67]. Similarly, TADs were maintained on the mouse Xi only in regions dense in escape genes in neural precursor cells [51].

Using a subset of seven genes that showed a consistent propensity to escape XCI in the Patski cell line, and in F_1_ mouse brain tissue, Deng *et al*. [47] found these to be located towards the outside of the 3D model of the Xi inferred from the contacts detected by Hi-C. A similar pattern was seen for CTCF, whose binding density was higher on the outside of the 3D structure of the Xi but not of the Xa, albeit mainly on one side of the structure. This is in line with previous findings by Chaumeil and co-workers that XCI escape genes tend to be located near the very outside of the inactivated interior of the Xi territory [47,68,69], and with findings showing a higher density of CTCF near escape genes [62]. By contrast, Lawrence's group proposed that both inactivated and escape genes are located at the periphery of the Barr body [70]. The preferential location of repeat elements is also controversial. Deng *et al*. reported that LINE1 (L1) repeat elements were preferentially located on the inside of the 3D model of the Xi structure. This is consistent with Mary Lyon's hypothesis on the role of LINE1 elements in XCI [71], and with previous findings that silent LINEs may participate in the assembly of a heterochromatic inner Xi compartment [47,68,72]. Contradicting this assertion is a study by Calabrese *et al*. [73], who reported that by FISH LINE1 elements were located externally on the Xi compared with silenced genes.

The relative distributions of genome features towards the outside or the inside of the 3D model of the Xi recall findings by Chadwick & Willard [22] who showed that during metaphase, the human Xi in RPE1 cells is marked by alternating bands of H3K9me3 or H3K27me3 that appear to co-localize to one or the other side of the condensed Xi during interphase. This suggested that the metaphase–interphase transition could be accompanied by Xi folding into two non-overlapping heterochromatin territories. Darrow *et al.* [52] confirmed this phenomenon by observing that a ChIP-seq signal for H3K27me3 during interphase largely overlaps the immunofluorescence pattern seen during metaphase, which indicates that the histone modification patterns actually persist between the two stages of the cell cycle with respect to linear genomic loci. Deletion of *DXZ4* in the RPE1 cell line not only resulted in a disruption of compartmentalization, as already mentioned above, but also disturbed the distribution of histone marks and the replication timing across a multi-megabase region around the deletion. By immunofluorescence, a band of H3K27me3 along the metaphase Xi seen in wild-type cells was lost in three independent *ΔDXZ4* clones and replaced by H3K9me3, with a concomitant switch from early to late replication, as determined by EdU incorporation [52].

## 5. Structure of the X-inactivation centre and spreading of *Xist* in three-dimensional space

A central mystery of XCI is how it comes to pass that *Xist* is upregulated from only one of the X chromosomes in the nucleus, seemingly at random. The *Xist* gene locus lies within a multi-megabase region of the X chromosome known as the X-inactivation centre (Xic), which harbours additional regulators of XCI, including the repressive antisense transcript, *Tsix* [74–77]. The findings by Nora *et al*. [30] introduced in §3 that first revealed TADs, were based on experiments conducted across a region spanning the Xic in the mouse. Thus, the Xic is itself partitioned into a series of TADs, along both the Xa and the Xi. Interestingly, Nora *et al.* found that the respective promoters of *Xist* and *Tsix* were located in distinct but adjacent TADs, along with their known positive regulators. Additionally, TADs were found to align with linear genomic features, such as H3K27me3, H3K9me2 and lamina-associated domains (LADs), as well as coordinately regulated gene clusters. Taken together, this suggests that TADs might serve to spatially segregate chromosomal neighbourhoods that are regulated in an opposing manner. In support of this, the authors found the disruption of a TAD boundary resulted in irregular chromosomal contacts and misregulation [30].

In a subsequent paper from the same laboratory, Giorgetti *et al*. [78] produced an ensemble of polymer models of the mouse Xic, which demonstrated that TADs represent the average contacts made by a diverse set of potential chromatin conformations within the ensemble of cells in the population, supported using single-cell FISH analysis. These results challenge the concept of stable long-range interactions between regulatory sequences and instead suggest that intra-TAD interactions are probabilistic in nature and based on interaction events that occur within a subset of cells at any one time. Indeed, a follow-up paper by the same group used the same modelling approach to demonstrate that the length of Xic DNA making up the *Tsix* and *Xist* TADs fluctuates between different conformational states relatively easily, implying that associated regulatory interactions are relatively unstable [79]. By measuring the motion of a site within the *Tsix* TAD using high-resolution live-cell imaging and simulating the temporal dynamics of the chromatin fibre, Tiana and colleagues predicted that *Tsix* TAD configuration changes occur on the order of tens of minutes, allowing for the possibility that enhancer–promoter sequences may go through multiple rounds of engagement and disengagement during the course of a single-cell cycle. Importantly, Giorgetti *et al.* [78] showed that the intrinsic fluctuations in the conformation of DNA fibres that make up the Xic TADs correlate with the transcriptional variation within the Xic, presumably due to the variability in distances between regulatory sequences. For example, *Tsix* and its putative regulator, *Linx*, both reside in the same TAD, but display opposing transcriptional states depending on the configuration of the TAD, with a more compact TAD structure corresponding to higher *Tsix* transcription levels and lower levels of *Linx*, and vice versa. The authors hypothesize that the two loci may compete for common regulatory sequences and that a clustered TAD may result in configuration of regulatory elements that favours *Tsix* transcription over *Linx*. Such stochasticity in expression potential within the TADs of the Xic may help to explain how it is that *Xist* is unlikely to be activated simultaneously from both alleles during differentiation.

The steps that follow the upregulation of *Xist* from the future Xi have been equally mysterious and the mechanism by which *Xist* proceeds to coat and inactivate the chromosome from which it is expressed is a very active area of investigation. RAP-seq (RNA antisense purification coupled to HTS) was developed with the specific aim of better understanding the mechanism by which *Xist* silences the Xi [4]. By using biotinylated RNA capture probes (antisense oligomers) on chromatin, RAP-seq allows one to determine the genomic locations that associate with an lncRNA of interest. With this technique, Engreitz and colleagues found that *Xist* RNA coating of the Xi does not proceed linearly from the *Xist* locus at the onset of XCI. Rather, *Xist* spreading initially depends on a proximity-mediated mechanism such that regions of the genome that are close to the *Xist* locus in 3D space are the first to be coated, even if many megabases away along the chromosome. In other words, the sites of early *Xist* coating are due to the inherent 3D conformation of the X chromosome. This result was confirmed when *Xist*, expressed from an ectopic site 50 Mb distal to the *Xist* locus or from an autosome, showed a similar dependence on 3D conformation for its initial pattern of spreading. Even though the study was conducted in a male mouse ESC line with an inducible *Xist* locus, which may not necessary reflect the endogenous process, it helps explain why *Xist* appears to require intermittent ‘way stations’ or ‘boosters’ early in the spreading process [71], and that, indeed, ‘a spreading model based on the linear sequence of the X chromosome might be an oversimplification’ [7].

## 6. Position of the inactive X chromosome in the nucleus

**Figure 2.**
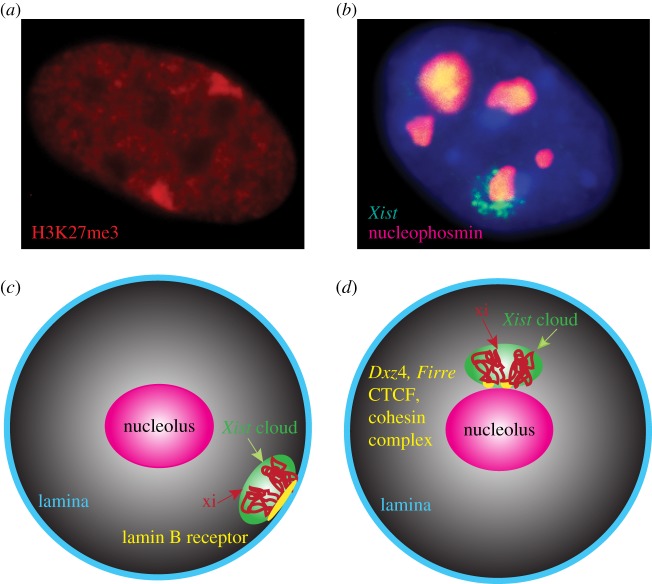
(*a*,*b*) Imaging of the Xi preferred locations near the nuclear periphery (*a*) or the nucleolus (*b*). (*a*) Example of a nucleus from an XXX individual after immunostaining for H3K27me3 shows two Barr bodies at different locations at the periphery of the nucleus. (*b*) Example of a nucleus stained with DAPI (blue) after immunostaining for nucleophosmin (red) and RNA-FISH for *Xist* to mark the Xi (green). Note that *Xist* RNA surrounds the nucleolus. (*c*,*d*) Hypothetical models of positioning of the Xi near the periphery which may be facilitated by the lamin B receptor (*c*) or near the nucleolus which may be facilitated by *Dxz4* and *Firre* lncRNA loci CTCF and cohesin (*d*). (Adapted from [96].)

By microscopy, the condensed Xi is often seen at the nuclear periphery (figure 2*a*) [12]. The second preferred position of the Xi is perinucleolar (figure 2*b*) [10]. These two locations are also preferred by other types of heterochromatin as shown in many different organisms [80,81]. The ‘Velcro’ effects of the lamina and nucleolus are only partially understood in terms of specific molecular features that attract heterochromatin and of specific genomic regions attracted to these locations [82]. The nuclear periphery comprises the nuclear membrane consisting of lipid bilayers traversed by the nuclear pores and the internal lamina, which contains lamin fibres that interact with chromatin at the LADs located throughout the genome [83–85]. The nucleolus interacts with the chromatin at the nucleolus-associated domains (NADs), and these interactions may be facilitated by proteins located at its edge (such as nucleophosmin) and by internal proteins (such as fibrillarin) that interact with ribosomal RNA genes [82,86,87]. Paradoxically, chromatin inside the nucleolus is highly active, whereas heterochromatin that surrounds the nucleolus is repressed. Repositioning of genes within the nucleus, for example to the nuclear lamina, can alter their expression, demonstrating the importance of nuclear location in gene regulation [88]. As discussed below, the roles of the lamina and the nucleolus in XCI have begun to be clarified by the identification of the molecular elements that facilitate association of the Xi to these specific nuclear compartments.

For instance, a new study has shown that recruitment of the Xi to the nuclear lamina is implemented via the Lamin B receptor (LBR), one of the proteins previously identified as a component of the *Xist* lncRNA–protein complex by quantitative mass spectrometry [89,90]. LBR is required to complete the process of *Xist* coating across the entire chromosome [89]. In combination with Engreitz's paper on the spreading of *Xist* (see §5) [4], this new study presents an appealing story arc to explain the process of XCI, as well as the importance of X-chromosome 3D structure. Furthermore, the LBR recruitment model seemingly fits well with the often-observed tendency of the Xi to be found towards the periphery of the nucleus (figure 2*a*,*c*) [28,91,92]. However, imaging using X painting probes has demonstrated that both Xa and Xi are often located close to the nuclear periphery [17]. Surprisingly, this same study also showed that the Xi had, in fact, a distinctly more internal nuclear position than the Xa, which was interpreted as possibly due to the Xa's elongated structure resulting in a higher frequency of apparent periphery touching [17]. An alternative explanation is that the Xa, which is upregulated to balance expression of X-linked and autosomal genes, may be preferentially located near nuclear pores, potentially to enhance RNA transport, similar to the situation in *Drosophila* [8,93]. An early study argued that the whole Xi may form a loop that would attach to the nuclear periphery via the telomeres tethered to the nuclear membrane close to each other [94], but such a structure has not been confirmed. Interestingly, in cells with three X chromosomes, the two Xis do not occupy the same exact location at the nuclear periphery but rather they remain separate, suggesting that the Xi does not occupy a specific single locus at the lamina (figure 2*a*). Furthermore, the location of the Xi varies between cell types. For example, in neutrophils where the nucleus is lobular, the Xi is preferentially located in a terminal lobe called ‘drumstick’ [95].

The second preferred location of the Xi is near the nucleolus. This location is cell cycle-dependent as shown in synchronized cell populations in which the Xi contacts the nucleolus in 80–90% of cells during mid-to-late S-phase, but not at G0, G1 and G2 [92]. Zhang *et al*. found that the Xi forms a ring around the nucleolus, which is enriched in Snf2 h, a protein required for heterochromatin replication (figure 2*b*). In addition, deletion of *Xist* resulted in loss of association of the Xi to the nucleolus, while insertion of *Xist* on an autosome caused it to associate with the nucleolus. This observation corresponds with the earliest characterization of the Xi as a ‘nucleolar satellite’ by Barr and Bertram [10], as well as with subsequent studies showing that the Xi tends to be localized at the nucleolus in both mice and humans [91,92]. In their aforementioned (in §2) light and electron microscopy study, Rego *et al.* [28] reported that the Xi made extensive contacts with the nuclear envelope and/or nucleolus, with several nuclei showing the Xi sandwiched between the periphery and the edge of a nucleolus.

In addition to *Xist*, other X-linked lncRNAs, including *Dxz4* and *Firre*, may help anchor the Xi to the nucleolus. Deng *et al.* [47] showed that the mouse *Dxz4* locus at the hinge between superdomains on the Xi was bound by both CTCF and nucleophosmin, a protein found at the periphery of the nucleolus. This is supported by the findings that NADs identified by purification of nucleoli followed by sequencing of associated DNA fragments are enriched in *DXZ4* in both human and mouse cells [47,86]. Another lncRNA locus, *Firre*, is also bound by CTCF and nucleophosmin specifically on the mouse Xi [96]. By fluorescence microscopy, both *Firre* and *Dxz4* on the Xi appear adjacent to the edge of the nucleolus in mouse fibroblasts, suggesting a role in tethering the Xi to the perinucleolar compartment [96]. However, the two loci rarely overlap, consistent with the apparent absence of a superloop detected by Hi-C in mouse cells as reported in two studies [47,51], but in contrast to another study that reported superloops between *Dxz4*, *Xist* and *Firre* [52]. *Firre* is expressed from the Xi, with evidence of multiple promoter sites specifically on the Xi, suggesting that this locus may produce short RNAs, similar to *Dxz4* [43,49,96–98]. Yang *et al.* [96] have shown that suppression of *Firre* expression by shRNA causes a decrease in association with the nucleolus and a loss of the repressive histone mark H3K27me3 on the Xi. Thus, *Firre* RNA may help maintain the heterochromatic state of the Xi. However, suppression of *Firre* RNA did not cause reactivation of genes on the Xi, again supporting the concept of multiple layers of control of gene silencing on the Xi. Taken together, these results indicate that *Dxz4* and *Firre* may serve as attachment sites allowing the nucleolus to act as ‘Velcro for heterochromatin’ with the hinge possibly representing an NAD, whose tethering to the nucleolus may govern the formation of a bipartite structure and the maintenance of the inactive state (figure 2*d*) [47,82]. Whether additional loci are implicated in the positioning of the Xi remains to be determined. By overlaying genomic feature density onto 3D models of the Xi, we observed that regions enriched for CTCF binding on the Xi tend to occur at the periphery of the 3D structure, suggesting potential additional attachment points [47].

## 7. Outlook

The role of the two superdomains in XCI-mediated gene silencing remains an open one. One possible explanation for the Xi's distinctive bipartite structure could be that it arises as an emergent property of XCI simply as a consequence of macrosatellite repeats serving as points of attachment to the nuclear periphery and nucleolus, mediated by CTCF binding. In this scenario, the bipartite structure would have no inherent function beyond that, which would help to explain the apparent lack of phenotype observed in *DXZ4* deletion mutants [47,51,52]. Whatever its ultimate role, it is somewhat difficult to reconcile the Xi bipartite structure observed with Hi-C with the clustering of distinct chromosomal bands of macroH2A and H3K9me3 seen at metaphase along the human Xi, which brings like chromatin types together in interphase [43,44,52]. Although the model proposed by Horakova and co-workers suggests a role for Xi-specific CTCF-bound *DXZ4* (along with *FIRRE* and *ICCE*) in segregating the H3K9me3 and macroH2A chromatin domains during interphase, it is not clear how this might result in the formation of the superdomains seen in Hi-C contact matrices. Deleting *FIRRE* and/or *ICCE* in conjunction with *DXZ4* might help to resolve this question. It would also be informative to perform finer-grained deletions within the hinge region (of the *DXZ4* locus itself, for instance) to see whether these might be sufficient to disrupt the bipartite structure of the Xi. It should be noted that significant mechanistic differences may exist between species: already, the content and condensation of superdomains are known to differ between human and mouse, and the existence of superloops originally found in human is controversial in mouse. Furthermore, the distribution of repressive histone modifications along the Xi differs between cell types and species [27]. Thus, 3D modelling of Xis from different species in relation to the distribution of heterochromatic features is warranted.

The bipartite structure may very well play an important role in XCI initiation, if not its maintenance. An important question in this regard is whether the hinge separating the two superdomains of condensation is flexible, which might allow each superdomain to be specifically positioned with respect to the nuclear compartments, such as the periphery and nucleolus. An alternative explanation for the hinge deletions having little phenotypic effect on gene expression could be that it reflects the fact that many layers of Xi repression are in place. It would therefore be interesting to investigate whether the loss of DNA methylation at CpG islands and/or the loss of repressive histone marks might lead to greater upregulation of previously silenced genes in conjunction with disruption of the Xi bipartite structure.

*XIST* and *FIRRE* interact with modified matrix proteins, such as Saf-A (hnRNP-U). This could eventually result in the Xi being tethered to the nuclear periphery or the nucleolus. Indeed, ‘the interaction of non-coding RNAs (ncRNAs) and the nuclear matrix might be a widespread phenomenon’ [99,100]. In addition, it has recently been shown that enhancer-derived lncRNAs may act as ‘molecular bridges that mediate spatial interactions’ between enhancers and promoters, or by perhaps reconfiguring the chromatin state to facilitate factor binding at promoters [101]. In a similar vein, perhaps certain X-linked lncRNAs, including *DXZ4* transcripts, may act as molecular bridges with respect to the nuclear matrix, in a manner analogous to *XIST*'s putative interactions with the nuclear scaffold. Along with RAP-seq (discussed in §5), the advent of the HiChIP technique, and closely related PLAC-seq, might help to shed light on the chromatin structure associated with particular ribonucleoprotein complexes [102,103]. Similarly, capture Hi-C methods allow for interactions specific to the X chromosome to be assayed for greatly reduced cost while improving resolution of interactions [104].

It should also be noted that the Barr body is not visible in all cells throughout their cell cycles, even though silencing as measured by protein electrophoresis is clearly highly stable in all cells [105,106]. Thus, absence of the Barr body in a given cell does not equate with absence of the Xi, which may be due to structural changes in the Xi during the cell cycle. This could be clarified using Hi-C on flow-sorted cell fractions and/or by single-cell Hi-C analyses. Single-cell Hi-C methods are starting to mature and could enable researchers to gain valuable insights into cell-to-cell variability in X-chromosome structure [107,108]. Live-cell imaging would also help determine how transient some interactions with nuclear organelles are.

The 3D tracing of TADs described in §3 goes some way towards bridging the often-disconnected worlds of X-chromosome imaging and HTS-based studies [53]. Researchers in the field should continue to pursue studies that integrate imaging and high-throughput biochemical techniques, especially with the advent of super-resolution microscopy [29,54]. Super-resolution imaging in conjunction with identification of specific sequences could help in gaining a better understanding of the unevenly condensed Xi structure and the mysterious channels inside it, which could potentially harbour specific functional elements such as genes that escape XCI. The very recent introduction of ATAC-see is another example of a newly developed method that begins to bridge the divide between imaging and biochemical methods in that it is a clever adaptation of a HTS method (ATAC-seq) for use in imaging studies [109,110]. This approach could help in gaining a dynamic view of the Xi structure during the cell cycle and in different cell types, something that is currently missing.

## Data accessibility

This article has no additional data.
